# Deprivation of loading during rat Achilles tendon healing affects extracellular matrix composition and structure, and reduces cell density and alignment

**DOI:** 10.1038/s41598-024-74783-w

**Published:** 2024-10-08

**Authors:** Malin Hammerman, Maria Pierantoni, Hanna Isaksson, Pernilla Eliasson

**Affiliations:** 1 Department of Biomedical Engineering, Lund University, Lund, Sweden; 2 Department of Biomedical and Clinical Sciences, Linköping University, Linköping, Sweden; 3 Sahlgrenska University Hospital, Department of Orthopaedics, Mölndal, 341 80 Sweden

**Keywords:** Histology, Immunohistochemistry, Collagen 1, Collagen 3, Elastin, Unloading, Experimental models of disease, Translational research, Preclinical research

## Abstract

Tendon healing involves mechanosensitive cells that adapt to mechanical stimuli through mechanotransduction, resulting in increased tissue strength. However, detailed insights into this process in response to different loads remain limited. We aimed to investigate how different loading regimes impact the spatial composition of elastin and collagens during Achilles tendon healing. Histological analysis was conducted on healing rat Achilles tendons exposed to (1) full loading, (2) reduced loading, or (3) minimal loading. Histological analysis included Hematoxylin & Eosin and immunohistochemical staining targeting elastin, Collagen 1, Collagen 3, and CD31. Our results showed that the impact of mechanical stimuli on healing tendons varied with the degree of loading. Unexpectedly, minimal loading led to higher staining intensity for collagens and elastin. However, tendons exposed to minimal loading appeared thinner and exhibited a less organized matrix structure, with fewer, less aligned, and more rounded cells. Additionally, our findings indicated an inverse correlation between angiogenesis and load level, with more blood vessels in tendons subjected to less loading. Tissue integrity improved by 12 weeks post-injury, but the healing process continued and did not regain the structure seen in intact tendons even after 20 weeks. This study reveals a load-dependent effect on matrix alignment, cell density, and cell alignment.

**Supplementary Information:**

The online version contains supplementary material available at 10.1038/s41598-024-74783-w.

## Introduction

Intact tendons are primarily composed of water (constituting up to 70% of their wet weight), extracellular matrix, and tenocytes. The extracellular matrix (ECM) is predominantly made up of type 1 collagen fibers (accounting for 60–85% of their dry weight), complemented by smaller amounts of other collagens, elastin, and proteoglycans^1^. Tenocytes are specialized fibroblasts within tendons that play a crucial role in synthesizing collagen and building a highly organized structure to create a tissue with substantial tensile mechanical strength. Despite their inherent strength, tendons are susceptible to ruptures, and among human tendons the Achilles tendon is most frequently injured^2–4^.

When tendons sustain a rupture, the intricate process of tendon healing is initiated, orchestrated by a complex interplay of cellular and molecular events. The healing of tendons is typically divided into three overlapping phases – the inflammatory, the proliferative, and the remodeling phases^5–7^. During the inflammatory phase, immune cells are recruited to the injured tissue. This is followed by the proliferative or reparative phase, a phase where tenocytes undergo proliferation and produce ECM. Collagen 3 is commonly produced first in large amounts, followed by an increased production of collagen 1^8–10^. Finally, the remodeling phase entails a dynamic reorganization of the ECM, facilitating the reconstruction of an organized collagen structure into fibrils and fibers. This remodeling phase is central for the reparative process during tendon healing. However, the repaired tissue rarely regains its pre-injury function, and scar tissue formation is often abundant^7,11,12^.

Mechanical loading can alter the tissue strength when introduced in all three phases of healing and is thereby an important extrinsic stimulus for healing tendons. Tenocytes are mechanosensitive, and can actively adapt to the mechanical environment, and this can occur through mechanotransduction in both intact and healing tendons. However, mechanical loading during early tendon healing can also alter the healing process through a secondary load-response, as it also increases the risk of microdamage, resulting in bleeding and prolonged inflammation^13–16^. Animal models can employ various methods to reduce loading during Achilles tendon healing. These methods are commonly utilized to investigate the importance of mechanical stimuli during different phases of healing, mainly on the important outcome from analysis of mechanical properties of the healing tendon tissue^7,17–22^. Structural mechanical properties of rat Achilles tendons, such as stiffness, peak force, and energy, typically converge within 4 weeks post-injury toward similar levels as in intact tendons, and unloading has shown to delay this recovery^20,23,24^. However, material properties, such as elastic modulus and stress, do not recover to the same extent during the same time span^20,23,24^. In addition, other studies indicate that mixed loading or intermediate loading might be more beneficial compared to complete unloading and full loading^12,24,25^.

To summarize, it is known that loading can stimulate and speed up the tendon healing process. However, the existing knowledge on differences between immobilization (minimal loading), reduced loading, and full loading is not complete. Few studies show different mechanical outputs between minimal loading, reduced loading, and full loading, but the biological mechanism behind the diverse response needs to be investigated. There is so far also limited data on the levels of elastin during tendon healing. Additionally, there is limited knowledge about how different levels of loading affect the spatial and temporal distribution of the fibrous component of the ECM in healing tendon tissues^20,26,27^. Hence, this study aimed to determine how varying levels of loading influence the ECM composition during early healing of rat Achilles tendons. Furthermore, we aimed to uncover the long-term recovery of the tendon tissue to gain insights into matrix remodeling and scar tissue formation in fully loaded tendons. This was done by staining for ECM proteins (elastin, collagen type 1, and collagen type 3), followed by qualitative and quantitative histological image analyses.

## Results

### Muscle atrophy and tendon size was load- and time-dependent

**Fig. 1:**
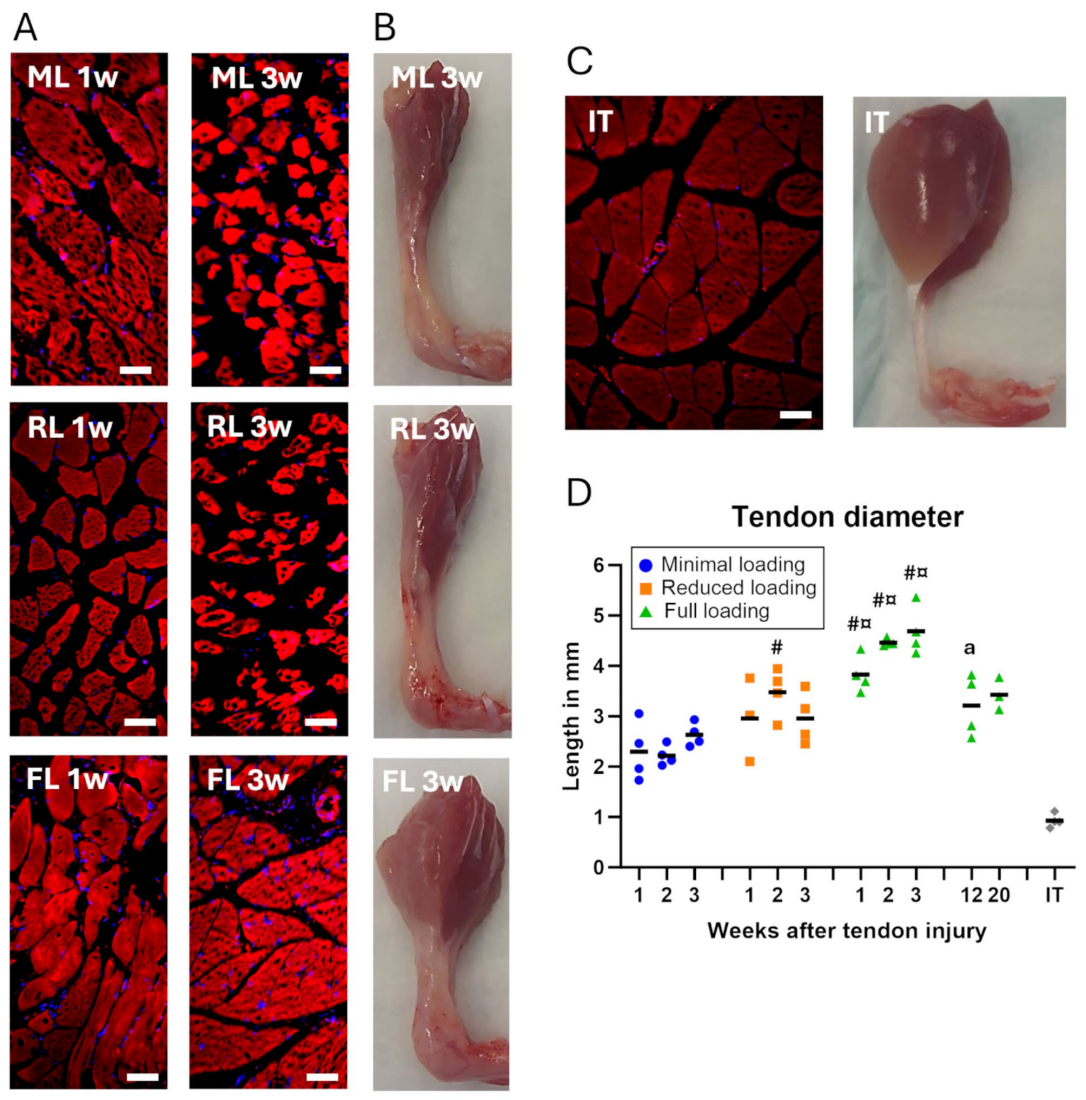
The effect of time and different loading conditions on the appearance of muscle and tendon tissue. (**A**) Muscle fibers in the calf muscle in different groups (FL: full loading, RL: reduced loading, ML: minimal loading) and time points, visualized by staining of elastin (red) and cell nuclei (blue). (**B**) Photographs of the Achilles tendon and calf muscle in different groups after 3 weeks of healing, taken from the side. (**C**) An intact tendon (IT) is shown as a reference. (**D**) Tendon diameter for different loading groups and time points, measured from histological sections. There was a significant effect of load levels during the early time-points and an effect of time between early and late healing. Scale bar 50 μm. # denotes a significant difference from minimal loading at the equivalent time point; ¤ denotes a significant difference between reduced and full loading; and ^a^ denotes a significant difference by time compared to 3 weeks.

Both models with different levels of unloading resulted in an expected and dramatic decrease in calf muscle size compared to full loading (Fig. 1B, Supp Fig. S1). Histologically, more muscle fiber degeneration was observed in both unloaded groups compared to full loading (Fig. 1A). Worth noticing, even the fully loaded group showed fiber degeneration since the Achilles tendon was fully transected. This was particularly seen at 1 week, when comparing to the muscles attached to intact tendons, whereas at 3 weeks the muscle fibers were more similar to the intact tendon muscle (Fig. 1A and C).

The size of the healing tendon was also affected by the load level, where calluses in the unloaded groups were smaller compared to full loading (Fig. 1B). This was confirmed by diameter measurements (*p* < 0.0001 for load level) on the histological slides, where both the minimal loaded and reduced loaded tendons had smaller diameter during the initial 3 weeks post-injury, compared to fully loaded tendons (40–50% and 22–37% less, respectively. Figure 1D).

### No apparent difference in spatial ECM distribution

The overall appearance, when comparing the different regions of the callus on both H&E and IHC staining’s showed no clear differences between the lateral side, the medial side, and center of the mid callus, at any time-points. This is also illustrated in the overall images showing the entire tendon (Supp Fig. S2-4^28^). Hence, further analyses are presented from the center image taken at the mid callus and at the stumps.

### Matrix structure and cell changes due to loading and time

**Fig. 2:**
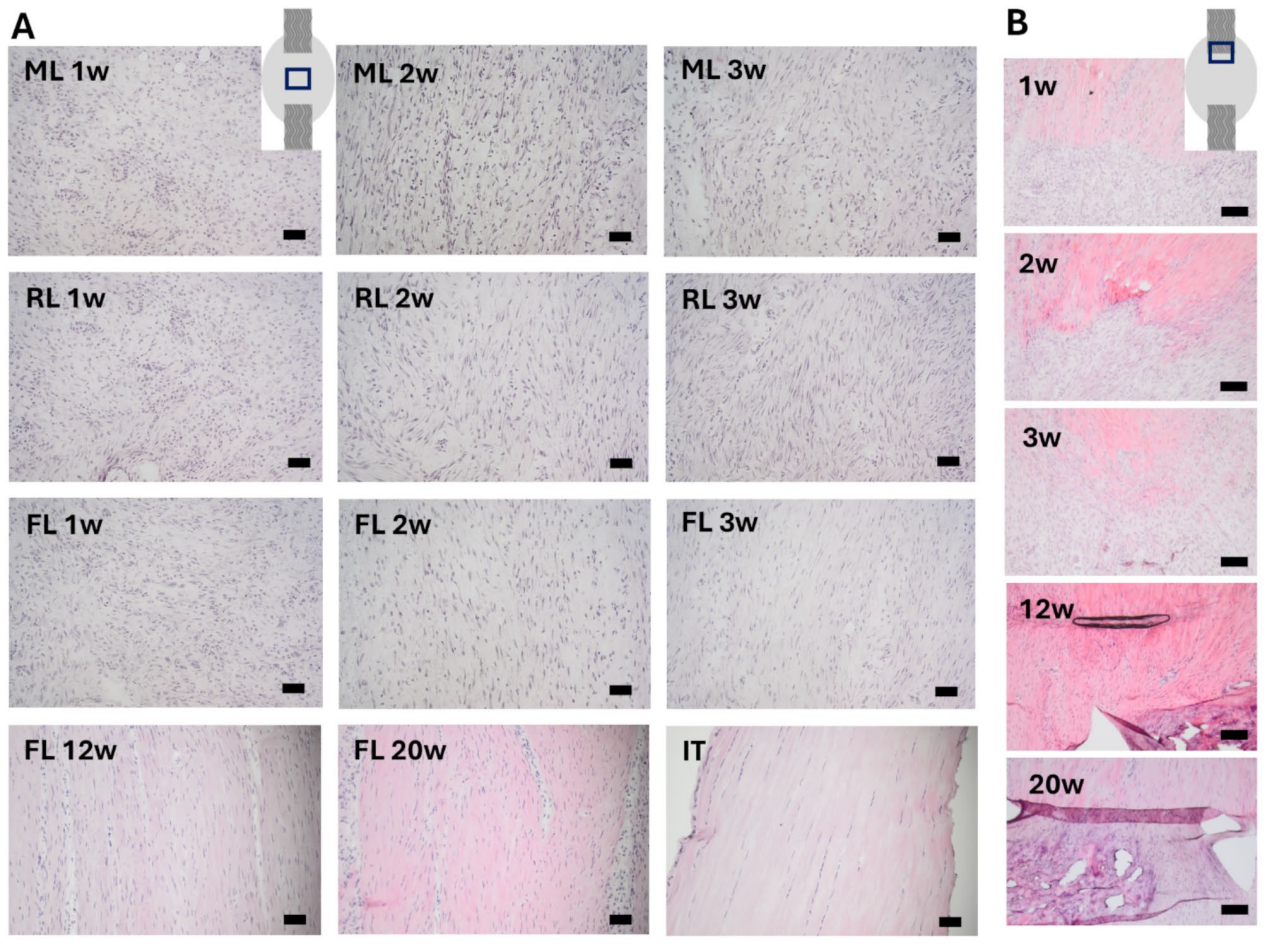
H&E staining of healing and intact Achilles tendons. (**A**) Healing or intact tendon tissue, taken in the middle of the Achilles tendon (location is shown in the insert). Healing tendons were studied at different time points after being exposed to one of three different loading conditions (ML: minimal loading, RL: reduced loading, or FL: full loading). An intact tendon is shown as a reference (IT). (**B**) Images taken at the tendon stump in FL rats, showing the progression of stump-integration over time (imaged location is shown in the insert). Scale bar 50 μm.

Descriptive H&E staining revealed that the healing tissue displayed an unorganized extracellular matrix across all groups at 1-week post-injury (Fig. 2A). At 2 weeks, the newly formed matrix began to align, and this was more pronounced in tendons with full loading and reduced loading compared to minimal loading (Fig. 2A). At 3 weeks, a more distinct aligned matrix and a higher matrix-to-cell ratio were found in the fully loaded group compared to the reduced and minimally loaded groups (Fig. 2A). Cells in 1-week healing tissue were mainly rounded in all groups, although fully loaded tendons also contained cells with a more spindle-shaped appearance and a higher degree of cell alignment compared to later time points (Fig. 2A).

The healing tissue close to the tendon stump appeared similar in all loading groups. The tendon stumps showed a distinct border towards the new healing tissue at 1 week (Fig. 2B). The healing tissue close to the distal stump appeared less compact and contained fewer cells compared to the tissue close to the proximal stump. After 2 weeks, the healing tissue was more integrated into the tendon stumps and the distinct border had disappeared (Fig. 2B). This was also seen for the tissue close to the distal stump that had an increased organization and contained more cells, resembling the tissue close to the proximal stump. After 3 weeks, the connection between the healing tissue and the stump was further improved, but the stumps were still clearly visible (Fig. 2B).

### Higher mean intensity of extracellular matrix protein staining in unloaded tendons

**Fig. 3:**
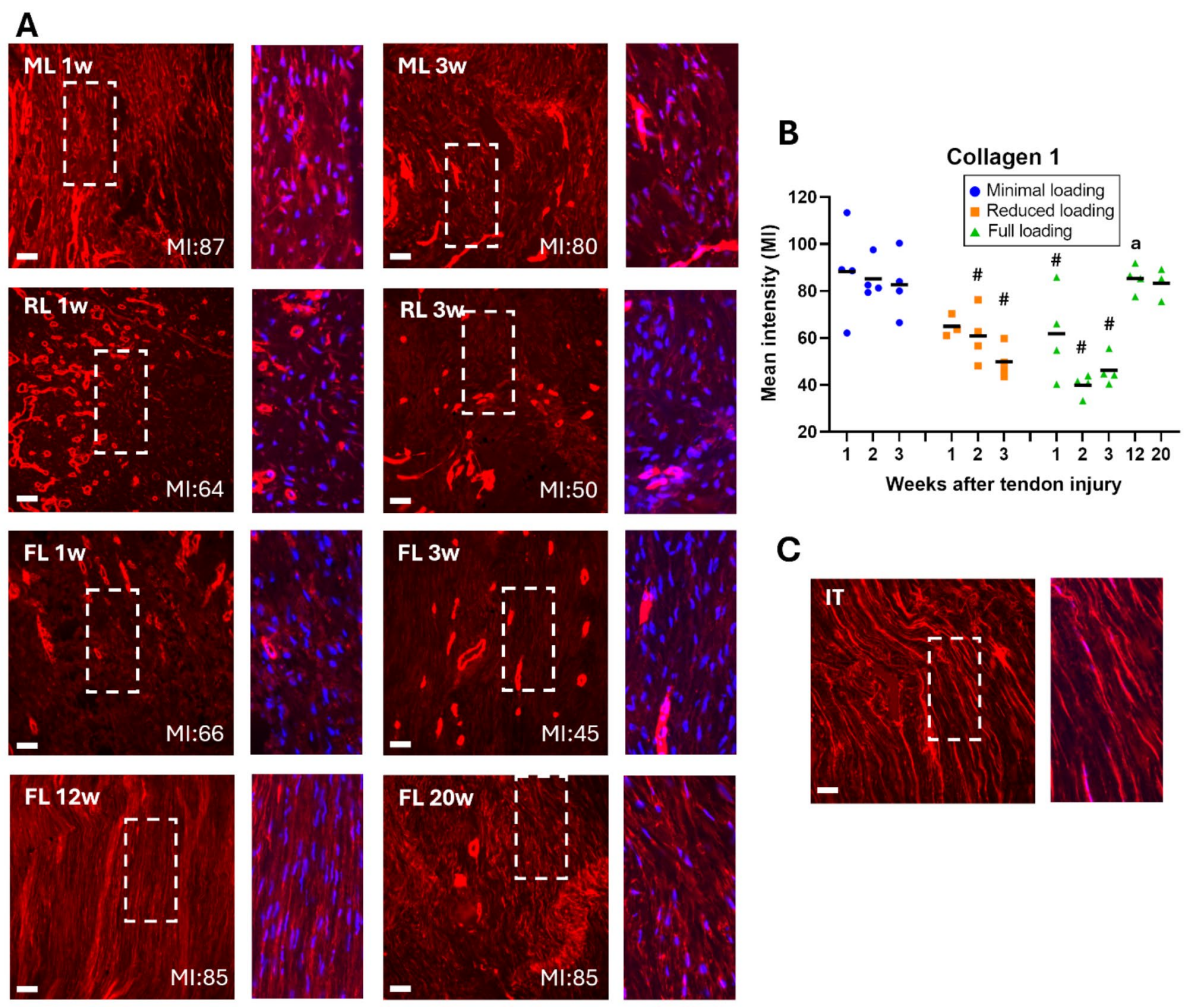
Higher mean intensity for collagen 1 in unloaded tendons. (**A**) Immunofluorescent staining for collagen 1 (red) in healing Achilles tendons at different time points after tendon injury and exposed to different loading conditions (ML: minimal loading, RL: reduced loading, or FL: full loading). Images were taken in the middle of the tendon tissue. Magnification of the marked areas show staining of collagen 1 (red) and cell nucleus (blue). (**B**) Mean intensity (MI) of collagen 1 for all individual tendons (dots) and mean (black line) for each group. (**C**) Intact tendon (IT), shown as a reference. There was a significant effect of load levels during the early time-points and an effect of time between early and late healing. Scale bar 50 μm. # denotes a significant difference from minimal loading at the equivalent time point; and ^a ^denotes a significant difference by time compared to 3 weeks.

**Fig. 4:**
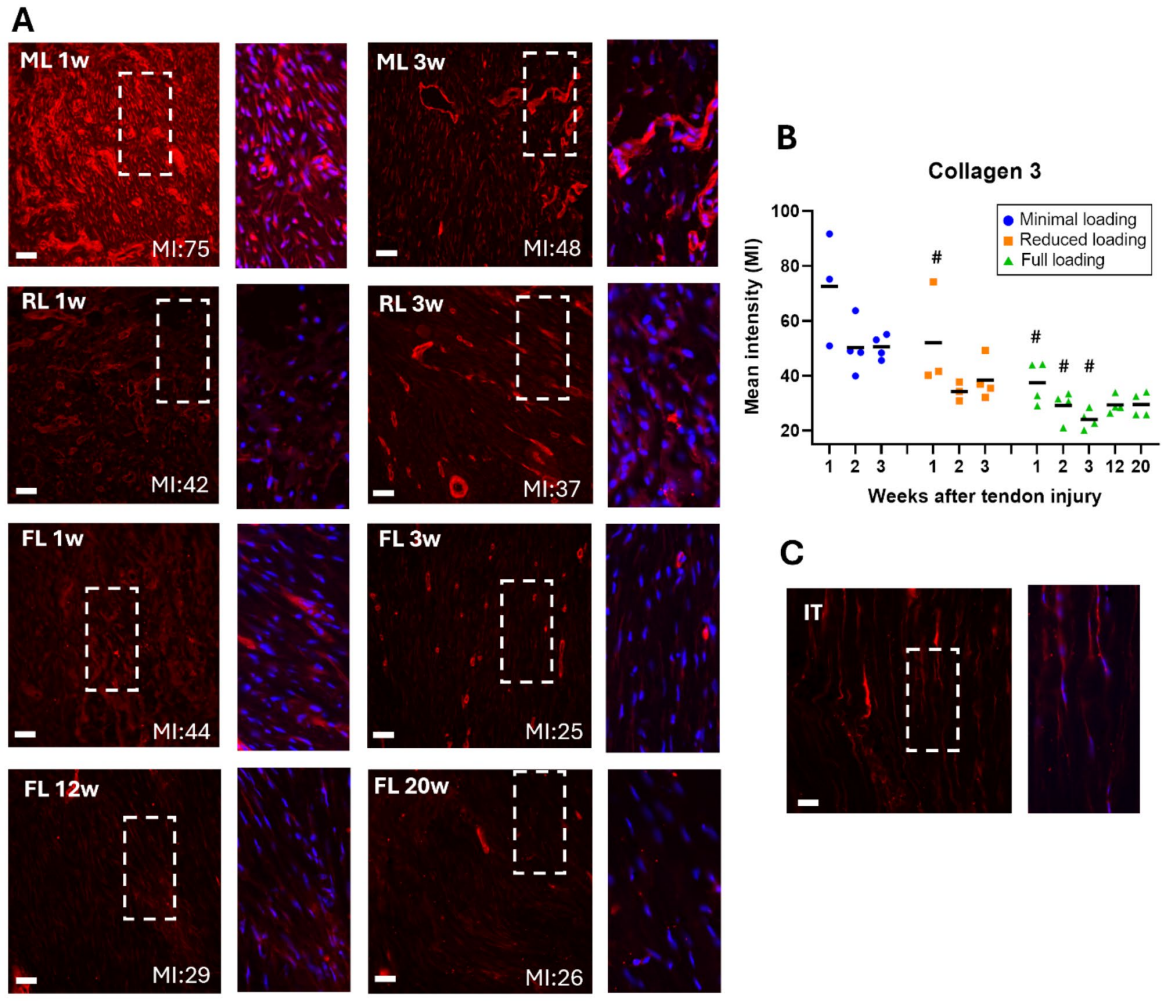
Higher mean intensity for collagen 3 in unloaded tendons. (**A**) Immunofluorescent staining for collagen 3 (red) in healing Achilles tendons at different time points after tendon injury and exposed to different loading conditions (ML: minimal loading, RL: reduced loading, or FL: full loading). Images were taken in the middle of the tendon tissue. Magnification of the marked areas show staining of collagen 3 (red) and cell nucleus (blue). (**B**) Mean intensity (MI) of collagen 3 for all individual tendons (dots) and mean (black line) for each group. (**C**) Intact tendon (IT), shown as a reference. There was a significant effect of load levels and time-points during the early tendon healing. Scale bar 50 μm. # denotes a significant difference from minimal loading at the equivalent time point.

**Fig. 5:**
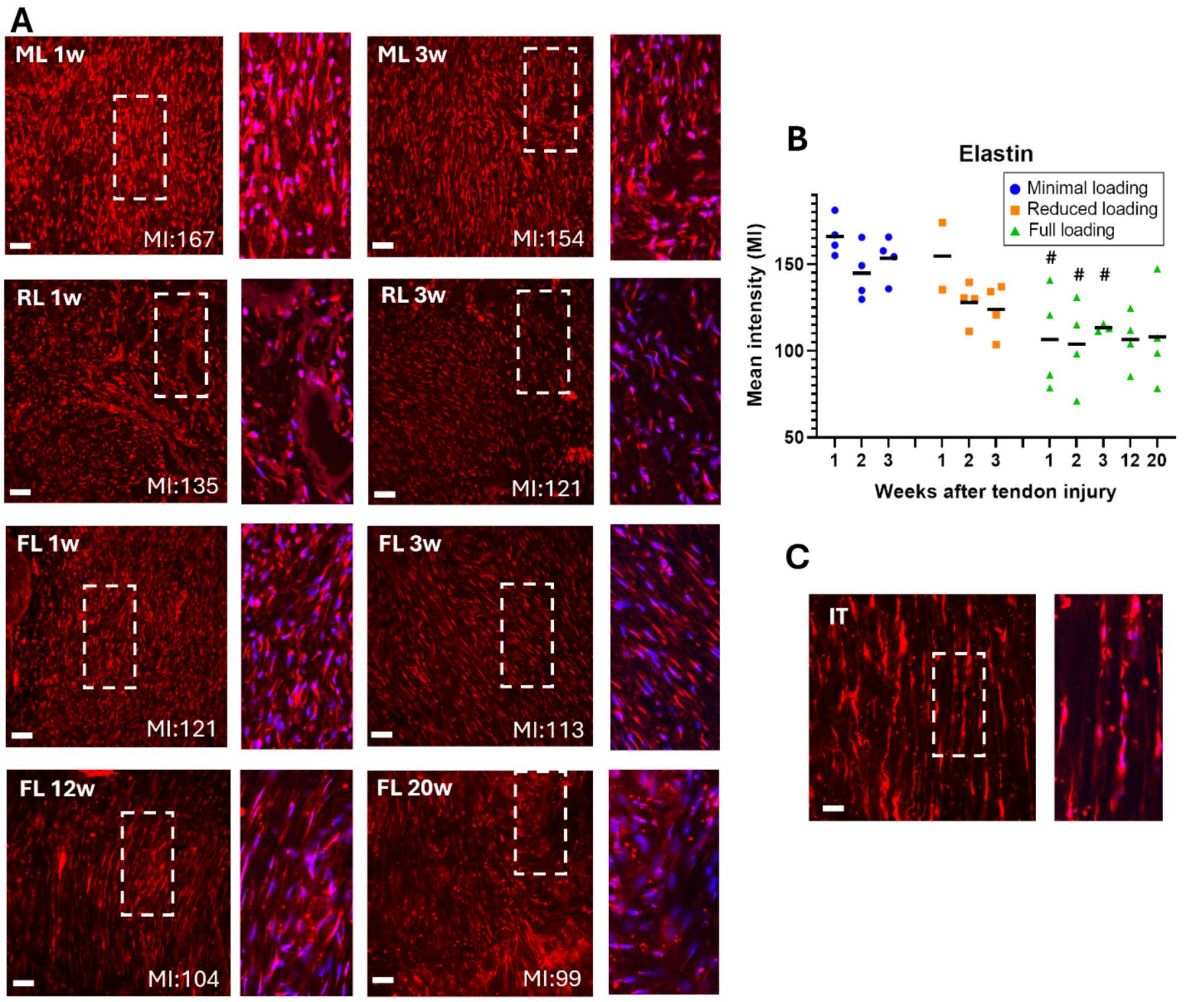
Higher mean intensity for elastin in unloaded tendons. (**A**) Immunofluorescent staining for elastin (red) in healing Achilles tendons at different time points after tendon injury and exposed to different loading conditions (ML: minimal loading, RL: reduced loading, or FL: full loading). Images were taken in the middle of the tendon tissue. Magnification of the marked areas show staining of elastin (red) and cell nucleus (blue). (**B**) Mean intensity (MI) of elastin for all individual tendons (dots) and mean (black line) for each group. (**C**) Intact tendon (IT), shown as a reference. There was a significant effect of load levels during the early time-points. Scale bar 50 μm. # denotes a significant difference from minimal loading at the equivalent time point.

The quantification of mean intensity, measured on IHC staining of the three matrix proteins (elastin, collagen 1, and collagen 3), indicated a load dependent response (*p* < 0.0001 for all proteins). The mean intensity of collagen 1 was significantly higher in minimal loaded tendons, in comparison to both reduced and full loading. This was seen across all three time points (Fig. 3, Supp Fig. S2). Minimal loaded tendons exhibited 43–114% higher mean intensity during the first three weeks compared to full loading, while reduced loaded tendons had 5–53% higher intensity compared to full loading. The mean intensities of collagen 3 (Fig. 4, Supp Fig. S3) and elastin (Fig. 5, Supp Fig. S4) were both significantly higher in minimally loaded tendons compared to fully loaded tendons. Collagen 3 exhibited a 72–113% higher mean intensity in minimal compared to fully loaded tendons, and reduced loaded tendons had a 17–58% higher intensity compared to full loading. For elastin, minimal loaded tendons had a 35–55% higher mean intensity, while reduced loaded tendons had 10–30% higher intensity compared to full loading.

### Loading can increase cell density and cell alignment

**Fig. 6:**
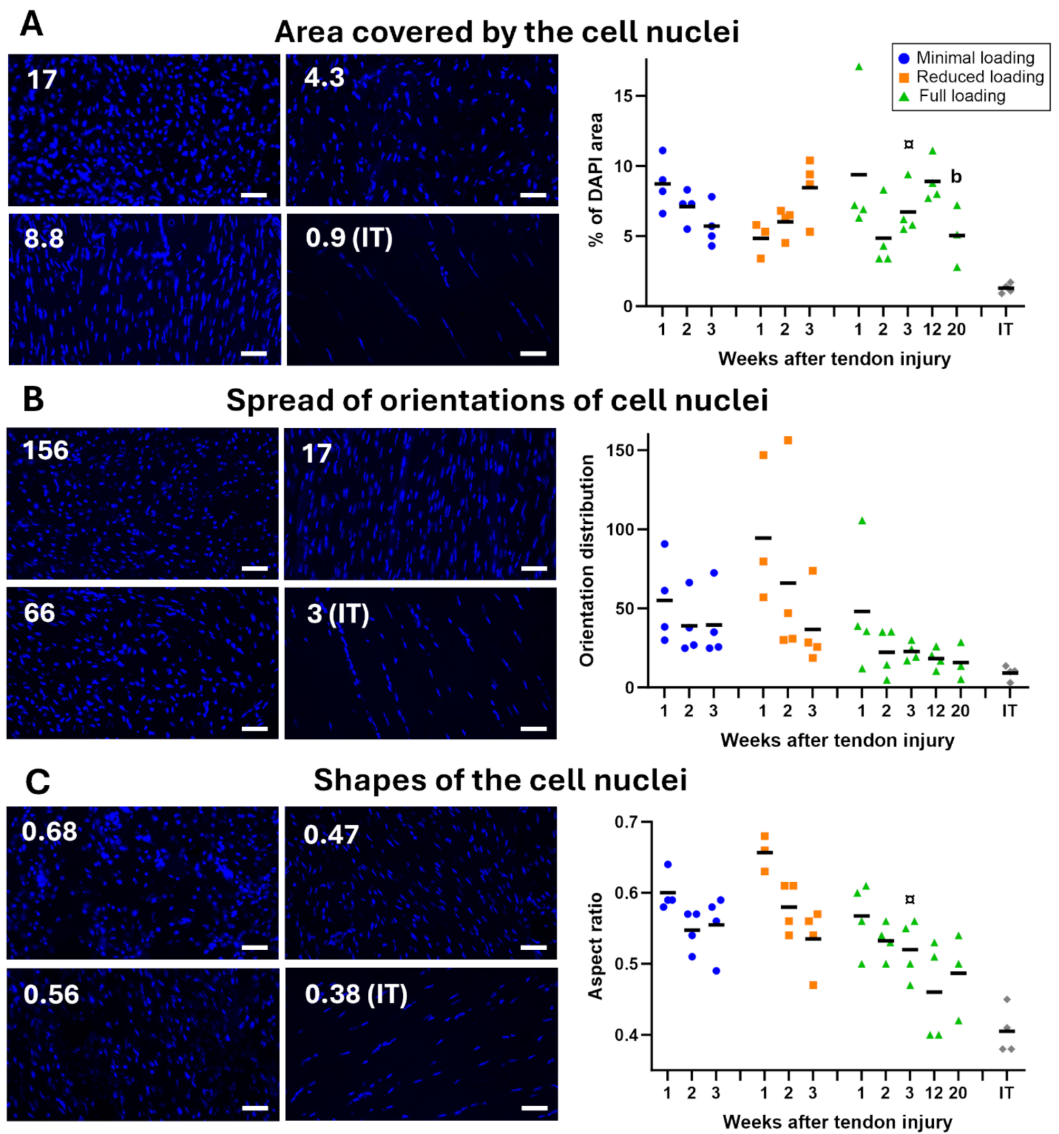
The effect of loading on cells. (**A**) Left: Representative images showing the range of values for cell nuclei area calculated as the percentage of area stained with DAPI. The lowest value was recorded from intact tendons (IT). Right: Percentual area covered by cell nuclei, plotted for all individual tendons (dots) and mean (black line) for each group. (**B**) Left: Representative images showing the range of values for the spread of orientations of cell nuclei. The lowest value was recorded from intact tendons (IT). Right: Orientation spread of cell nuclei, plotted for all individual tendons (dots) and mean (black line) for each group. There was a trend to difference of load levels and time-points during early tendon healing (*p* = 0.056 and 0.066, respectively). (**C**) Representative images showing the range of values for cell nuclei shape. The lowest value was recorded from intact tendons (IT). Right: Nucleus cell shape, plotted for all individual tendons (dots) and mean (black line) for each group. There was a significant effect of both load levels and time-points during the early tendon healing. Scale bar 50 μm. ¤ denotes a significant difference from reduced loading at the equivalent time point; and ^b^ denotes a significant difference by time compared to 12 weeks.

Quantitative analysis of DAPI stained images (Fig. 6) showed that there were differences between the loading groups in cell alignment (*p* = 0.056) and cell shape (*p* = 0.0003). Furthermore, cell density was high in all healing tendons (~ 5–10%) compared to intact tendons (~ 1%, Fig. 6A). Both cell alignment and shape of the nuclei elongated over time (*p* = 0.066 and 0.0013, respectively) (Fig. 6B & C). However, the highest alignment (less spread of angles) and the lowest value in cell shape (more spindle-like shape) was found in fully loaded tendons at 3 weeks post-injury (Fig. 6B & C).

### Normalization to tendon diameter partly diminished differences between groups

**Fig. 7:**
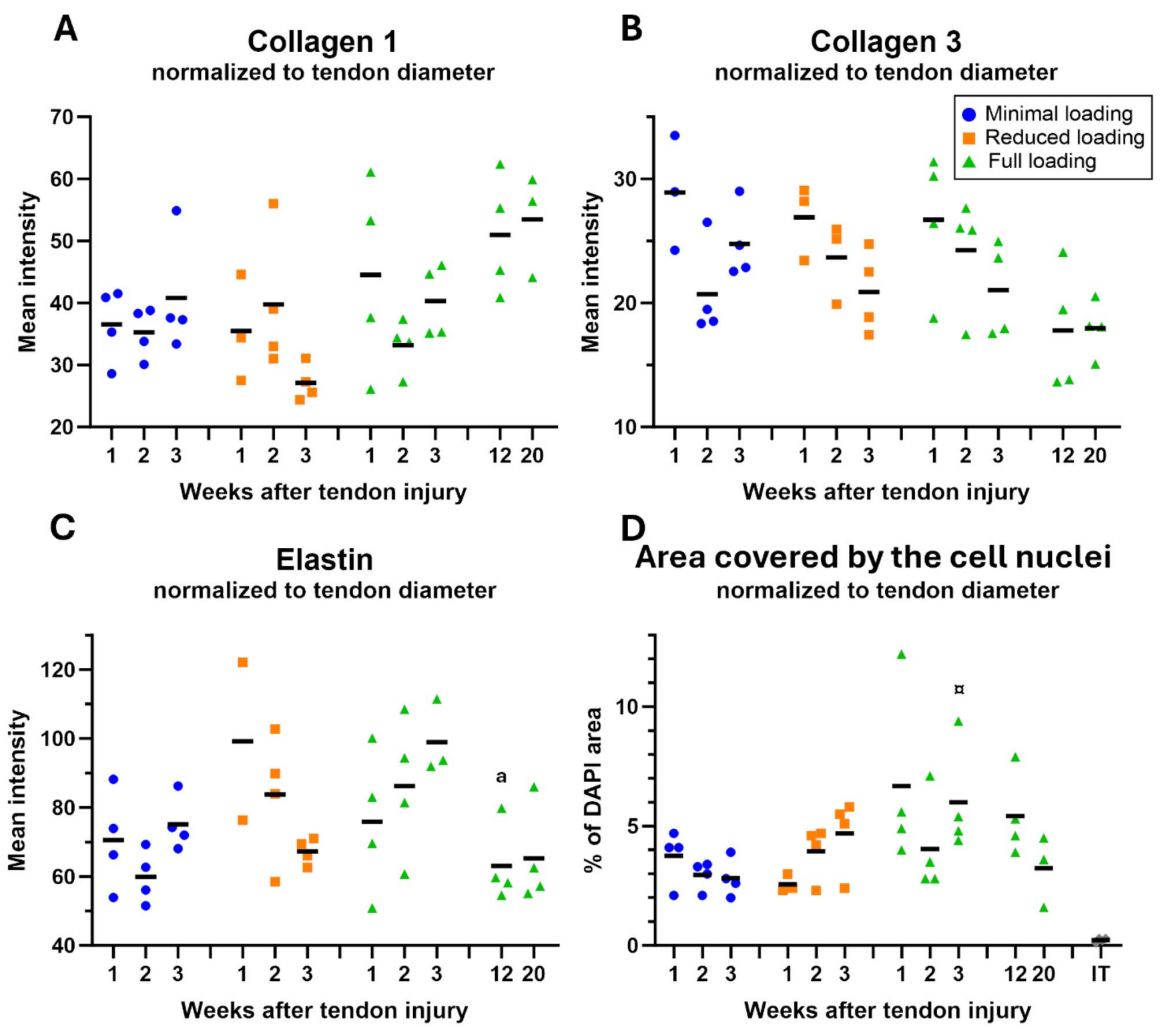
Normalization to tendon diameter decreases differences between groups regarding mean intensity and cell nuclei area. (**A**-**C**) Mean intensity of collagen 1, collagen 3, and elastin normalized to their tendon diameter. There was a significant effect of load levels on collagen 3. (**D**) Percentual area covered by cell nucleus normalized to tendon diameter. Dots represent individual values for each tendon and the black line represents the mean for each group. ¤ denotes a significant difference from reduced loading at the equivalent time point; and ^a^ denotes a significant difference by time compared to 3 weeks.

Normalization by tendon diameter was performed, as a simplified way to assess the effect of size, since minimal and reduced loaded tendons were substantially thinner than fully loaded tendons. It led to reduced differences between the loading groups in mean intensity of the ECM protein staining’s (Fig. 7A-C). The mean intensity for collagen 3 had still a significant difference for load levels (*p* = 0.013), elastin had a trend (*p* = 0.067), while the effect of load on collagen 1 diminished. Cell density was also normalized to tendon diameter which resulted in a cell density between 3 and 7% in healing tendons, whereas intact tendons had a cell density of 0.1% (Fig. 7D). Interestingly, fully loaded tendons had a cell density between 4 and 7%, whereas unloaded tendons had a cell density between 3 and 5%.

### Blood vessel regeneration

**Fig. 8:**
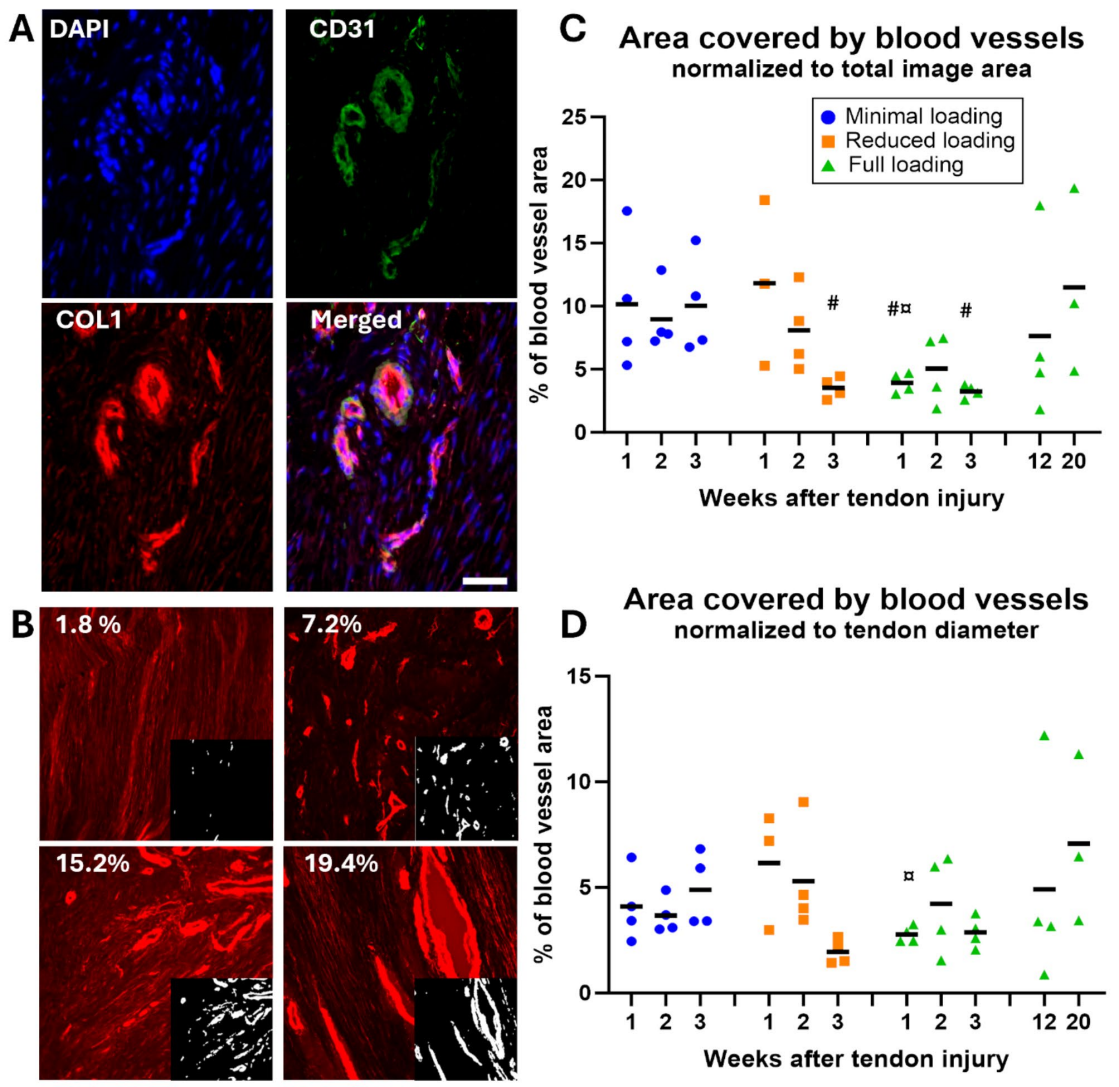
Blood vessel regeneration in healing tendons. (**A**) Areas with high intensity of collagen 1 staining in healing tendons appeared as blood vessels and were confirmed by CD31 antibodies, a marker for endothelial cells. Scale bar 50 μm. (**B**) Representative pictures showing the range of values for blood vessel areas, based on collagen 1 (red) staining. Areas detected as blood vessels (white) by image analysis are shown in the smaller inserts to the right. (**C**-**D**) Percentage of area covered by blood vessels, plotted for all individual tendons (dots) and mean (black line) for each group, before (**C**) and after (**D**) normalization by tendon diameter. There was a significant effect of load levels on the non-normalized data. # denotes a significant difference from minimal loading at the equivalent time point; and ¤ denotes a significant difference from reduced loading at the equivalent time point.

Staining of ECM proteins (collagen 1 and 3) revealed numerous blood vessels throughout the healing process, and this observation was confirmed by CD31 staining (Fig. 8A). Unloading showed a higher blood vessel density (8–12%) compared to full loading (3–4%) at 1- and 2-weeks post-injury (Fig. 8B & C), and there was a significant effect of load levels (*p* = 0.001). After normalization by tendon diameter, differences between unloading and full loading were reduced (Fig. 8D).

### Tissue regeneration and remodeling continues without restoring intact tissue integrity

At later time points (12- and 20-weeks post-injury) changes in matrix composition were evident. Matrix alignment and spindle-shaped appearance of cells in the healing tendon tissue became more pronounced, maintaining similarity between 12 and 20 weeks (Figs. 2A and 6C). However, despite these alterations, the well-organized structure and low cell density observed in intact tendons (Fig. 2A) were not reached even after 20 weeks of healing.

Mean intensity of collagen 1 increased with 46% between week 3 and 12 (*p* < 0.0001), and these elevated levels remained consistent until week 20 (Fig. 3, Supp Fig. S5). Conversely, mean intensity of collagen 3 remained similar from 3 weeks and onwards (Fig. 4), while mean intensity of elastin was similar across all time-points (Fig. 5). Upon normalization to tendon diameter, collagen 1 demonstrated a 27% increase in mean intensity between week 3 and 12, whereas collagen 3 and elastin showed a decrease of 19% and 36%, respectively (Fig. 5). The decrease in elastin levels between 3 and 12 weeks was significant (*p* = 0.013). Mean intensity thereafter remained stable for all three proteins until week 20 (Fig. 7B-C). Concurrently, cell density decreased between 12 and 20 weeks (*p* = 0.056) (Figs. 6 and 7).

The stumps were more integrated into the healing tissue at 12- and 20-weeks post-injury, compared to 3-weeks healing tendons, but still distinguishable (Fig. 6B). Heterotopic ossification was observed at both ends, with generally larger heterotopic ossification at the proximal stump compared to the distal stump (Fig. 6B).

## Discussion

To increase our understanding of how mechano-regulatory mechanisms play a role during tendon healing, and ultimately gain knowledge on how diverse loading regimens can be used to promote recovery, we investigated the relationship between different levels of mechanical loading and spatiotemporal structural changes in three important matrix proteins (Collagen 1, 3, and elastin) in healing tendon tissue. Overall, we found that the impact of mechanical stimuli on healing tendons varies depending on the level of loading. Somewhat counter-intuitive, minimal loading resulted in the highest staining intensity for collagens and elastin, as assessed by immunohistochemistry. However, tendons subjected to minimal loading were simultaneously thinner and exhibited a less organized matrix structure. These minimally loaded tendons also had fewer cells, which were less well-aligned and more rounded. Moreover, our results show an inverse relationship between angiogenesis and the level of loading, as more blood vessels were detected in tendons subjected to less loading.

Mechanical loading during early stages of tendon healing has been shown to positively affect the structural mechanical properties of healing tendons, as demonstrated in both animal models and patients^20,23,26,29,30^. In this study, three different loading conditions (minimal, reduced, and full loading) were implemented. Full mobility was permitted to rats in the fully loaded group following tendon injury. Those rats exposed to reduced loading received Botox in their calf muscle to inhibit active muscle contraction, yet they could still limp on their leg leading to partial indirect loading. Conversely, rats exposed to minimal loading also received Botox along with an orthosis, to further restrict indirect loading. These load levels were selected from previous studies, by Andersson et al. (2012) and Hammerman et al. (2018), where we showed that minimal to reduced loading was sufficient to improve material properties of the healing tissue at 8 days post-injury^23,24^. Their main finding though was that full loading primarily results in increased tendon volume, leading to a thicker and stronger tendon.

Large spatial distribution was not observed when comparing the lateral side, medial side or the center of the mid-callus, for any of the three proteins studied. This observation diverges from results reported by Sasaki et al. and Khayyeri et al^26,31^, although other results in this study were similar to previous ones. Sasaki et al. revealed by electron microscopy, a random arrangement of collagen fibers at 1 week, transitioning to an axial arrangement by 2 weeks, facilitating connection between the repair site and the tendon stump^31^. Similarly, we noted a distinct boundary in the healing tissue near the tendon stump at 1 week, with increased integration of the stumps into the healing tissue by the 2nd week, regardless of loading exposure. Khayyeri et al. investigated the full tendon callus, by small angle x-ray scattering, projecting the scattering through the 3D callus onto a 2D detector^26^. Additionally, we recently examined the 3D structure and organization of both collagen fibers and fibrils^27,32^. In those studies, we found that stump remodeling was delayed in minimal loaded compared to fully loaded tendons, a finding seemingly at odds with the current study’s results. However, we previously noticed that integration of collagen fibers between the healing tissue and the stump seems to occur predominantly from the side of the stumps, rather than from the vertical transection. In the current study, our focus was only on the vertical transection of the stump. It is therefore possible that differences between loading groups could be more pronounced, when considering integration from the side of the stumps, which needs to be further evaluated.

Full loading during early stages of healing appears to promote cell proliferation or increase cell migration, as evidenced by a larger area covered by cell nuclei in the fully loaded group compared to the minimally loaded group at 3 weeks post-injury. Cells in fully loaded tendons also exhibited higher alignment. Additionally, improved organization of the ECM was observed with increased loading as seen by H&E staining, a distinction that became particularly evident at 3-weeks post-injury. These observations contradict previous findings, where no significant difference in cellularity was observed between two loading regimens, and where the immobilized group exhibited higher fiber organization, an elevated collagen type 1:3 ratio, and increased echogenicity compared to a group engaged in early treadmill running^12^. Regarding angiogenesis, we observed that minimally loaded tendons exhibited a higher density of blood vessels. During early stages of healing, blood vessels were small and numerous. As the healing progressed, the number of blood vessels decreased, but their size increased, which is reflected in the increase in the area covered at the later time points, specifically in the 12- and 20-weeks tendons.

Our quantitative image analysis revealed that tendons exposed to minimal loading exhibited a higher mean intensity for both collagens and elastin compared to fully loaded tendons. The most significant differences were observed between tendons with minimal and full loading, indicating that matrix proteins respond to unloading in a dose-dependent manner, with reduced loading correlating with a higher density of matrix proteins. Contradictory, we have previously shown that gene expression of collagen 1 and 3, at 5 days post-injury, are instead increased by loading in a dose-dependent manner^24^, and similar results have also been shown in other studies^33^. However, the literature findings, regarding the effect of unloading or mixed loading on collagen 1:3 ratio, is generally not consistent^20^. Our findings are consistent with a previous study by Freedman et al., that demonstrated slightly elevated levels of collagen 1 and 3 in an immobilized group, compared to a group with early return to activity^25^. Moreover, six weeks of immobilization was found to result in a smaller cross-sectional area of the tendon, together with an increase in tendon echogenicity and collagen alignment^12^. Although mechanical loading has a strong effect on tendon healing, immobilization angle has also been shown to influence collagen alignment, where more dorsiflexed immobilization leads to better alignment^34^.

Elastin levels have been previously investigated in intact tendons, and there is currently one study showing higher levels of elastin in healing tendons with full loading compared to intact tendons^35^. However, the effect of reduced load levels has not been studied before. In this study, we showed intense elastin staining compared to intact tendons. The intense staining was observed across all loading groups and is consistent with the previous study on fully loading healing tendons^35^. Our results showed that minimal loaded tendons exhibited the highest mean intensity, especially at 3-weeks post-injury. The results for 12 and 20 weeks showed that the elastin content was stabilized at similar levels to early healing. It is conceivable that elastin acts as a protective mechanism against re-injury, as it has been shown to be important for the viscoelastic behavior^35–37^, and possibly enhancing tendon compliance to mitigate further injuries during the healing process.

Our results suggest that the proliferative phase during tendon healing in rats commences within the first week after injury, marked by an increased production of collagen 3, which appears to peak around 1 week, regardless of loading exposure. Previous studies on collagen 3 levels have shown decreasing levels during the first 8 weeks, but with different peak time-points^9,38–40^. Our findings indicate that the initiation of collagen 1 production occurs approximately 2–3 weeks after injury, particularly in fully loaded tendons, while unloading may delay this process. Collagen 1 content seems then to peak between 3 and 12 weeks, signifying the transition to the remodeling phase. Previous studies have demonstrated a progressive increase in staining intensity for collagen 1 during the initial 8 weeks post-injury^9,38^, while da Silva et al. showed increasing collagen alignment during the initial 17 weeks, but with a simultaneous decrease in collagen 1 staining^41^. Our results further demonstrate that, between 3 and 12 weeks of healing, some parameters are improved in the healing tendon tissue, exemplified by the increase of collagen 1. As healing progresses, the organization of the cells in the healing tissue improves, with a more elongated appearance and greater alignment. Changes between week 12 to 20 are more subtle, characterized by a decrease in cell density. However, cell density in 20-weeks tendons remains higher compared to intact tendons. Importantly, this final phase persists even beyond 20 weeks of healing, as none of the extracellular matrix proteins regain the same tissue structure observed in intact tendons.

As our results showed higher mean intensity in unloaded tendons, i.e. higher density of these three matrix proteins, one might expect that unloaded tendons are stronger. However, our previous study has indicated the opposite, associating more loading with stronger tendons^24^. This is likely a result due to size, as the newly formed tendon tissue in fully loaded tendons tends to be thicker than in unloaded tendons in a dose-dependent manner. Therefore, to account for these size differences in callus tissues seen between the three loading groups, mean intensity of the three matrix proteins was normalized to tendon diameter. Normalization by tendon diameter changed the results so that the disparities between groups notably diminished or even exhibited opposing results, implying that unloading has a restricted influence on the production rate of these matrix proteins during early healing. However, the fact that fully loaded tendons have a thicker callus indicates that these matrix proteins are deposited in wider volumes compared to reduced and minimal loaded tendons, which instead seems to have a higher concentration of these proteins in a smaller area. Differences between the three loading groups regarding cell density, became instead more evident after normalization by tendon diameter, where more loading resulted in higher cell density.

At both 12 and 20 weeks of healing, the old tendon stumps remained visible and substantial areas of heterotopic ossification were observed, typically near the tendon stumps. In rats undergoing Achilles tendon healing, heterotopic ossification is a common aberrant process in which pathologic bone is deposited in the soft tendon tissue^42^. This process was documented to initiate at the distal stump during the early inflammatory phase^26,43–45^. Over time, additional deposits formed in the proximal stump, and sequentially in the callus tissue^43,44^. By 20 weeks, these deposits had merged into large formations, occupying on average ~ 8% of the tendon volume^43^. Previous studies have suggested a connection between angiogenesis and pathological ossification, indicating that increased vascularization precedes heterotopic ossification^46,47^. However, a higher density of blood vessels at the stumps were not observed in this study, and the fact that blood vessel density increased with time can only partially explain the ossification pattern seen in this study, which first started in or around the stump, and proceeded in the gap tissue only later.

While histological analysis is a valuable tool in tendon research, it is essential to recognize and address limitations associated with a small sample number, 2D quantification based on a small area, and, in this study, tendon diameter measurements from histological slides. One limitation of the current study is the lack of mechanical data. However, we have presented mechanical testing in several previous studies with the same type of loading regimens or time points^23,24,26^. Furthermore, cell density was measured as the area covered, where individual nuclei were not counted. This provided an automated non-investigator dependent method for quantification. However, differences in cell size or shape could influence these results. The area covered by blood vessels was determined from the collagen 1 staining, not from a specific vessel marker such as VEGF or CD31. However, CD31 was used as a marker for endothelial cells to confirm co-expression. Additionally, this study solely focused on the fibrous component of the ECM, excluding other important constituents such as proteoglycans and water content. Thus, our focus does not provide a complete understanding of how tendon composition and function develop over time and in response to altered loading environment. We used Botox injections to alter loading and this might cause muscle damage and trigger an inflammatory response, which could influence the tendon healing process. Furthermore, the cross-talk between muscle and tendon tissues was not considered in this study. However, the potential for an acute damaging effect from the Botox is limited, as the drug was administered 4 days before the tendon transection was performed. Only female rats were used in this study as they have a lower growth rate than male rats which makes them more suitable for the chosen immobilization model (a custom-made boot in one size). However, previous research has shown sex-based differences, with female rats exhibiting more collagen type 3 at the injury site compared to male rats. Additionally, female rats demonstrate differences in the recovery of mechanical properties, such as viscoelasticity, and better muscle fiber preservation^48^. The growth rate of the rats also limits the use of the boot for long-term experiments (12 and 20 weeks), where only fully loaded tendons were studied due to concerns about prolonged unloading and its potential impact on animal welfare.

## Conclusions

Mechanical loading during early stages of tendon healing has positive effects on the healing tendon tissue. It is already known that increased loading results in larger and stronger tendons, but our study demonstrates that loading also increases cell density, and more importantly cell and matrix alignment. This study showed that elastin levels during tendon healing are high in healing tendons.  Our results regarding the effect of loading on the formation of collagen 1, collagen 3, and elastin during early tendon healing is contra-intuitive. On one hand, the staining intensity was highest in the minimally loaded tendons, but when accounting for size, the total amount of proteins seems to be higher in the fully loaded tendons. Healing tendons exposed to minimal or reduced loading are thinner and shorter and show a high density of these proteins in their callus, whereas fully loaded tendons are thicker and longer, and show a lower density of these proteins in their callus. As the healing progresses, the healing tendon tissue remodels as cell and ECM increase their alignment, cell density decreases, collagen 1 content increases, and content of collagen 3 and elastin decreases. Most of these improvements have already occurred at 12 weeks post-injury, but the healing process is still ongoing in 20-weeks tendons, as the ECM has not regained the same structure observed in intact tendons.

## Materials and methods

### Study design

Histological analysis was performed on healing Achilles tendons from rats that had been exposed to different levels of loading. All rats were surgically injured on their right Achilles tendon, and divided into short-term healing (1-, 2-, and 3-weeks, *n* = 39, Table 1) or long-term healing (12- and 20-weeks, *n* = 8, Table 1). The rats were randomized by lottery. Rats for short-term healing were divided in three groups; full loading (FL: free cage activity, *n* = 4/week, Table 1), reduced loading (RL: paralysis of the calf muscle with Botox, *n* = 4/week, Table 1), and minimal loading (ML: Botox combined with joint immobilization using a steel-orthosis, *n* = 5/week, Table 1)^24^. Rats for long-term healing were all subjected to full loading (free cage activity, *n* = 4/week, Table 1). Three rats from the minimal loading group were excluded due to wounds on the paw caused by the steel-orthosis. These animals were euthanized at 7-, 9-, and 10-days post-surgery. In total, 47 tendons undergoing healing were collected for histological analysis, and 4 rats were used to collect intact Achilles tendons, to be used as a reference.

**Table 1:** Study set-up and sample size in experimental groups. Intervention abbreviations: ATT = Achilles tendon transection, FCA = free cage activity, PCM = paralysis of calf muscle, SO = steel- orthosis. End point = weeks after ATT.

Group	ATT	FCA	PCM	SO	End point (Week)	*N*
Full loading (FL)	X	X	-	-	1, 2, 3, 12, 20	20 (4/week)
Reduced loading (RL)	X	X	X	-	1, 2, 3	12 (4/week)
Minimal loading (ML)	X	X	X	X	1, 2, 3	15 (5/week)
Intact tendon (IT)	-	X	-	-	-	4

### Animal model

Female Sprague-Dawley rats, specific-pathogen free (11–12 weeks, weight 299 ± 15 g), were bought from Janvier (Le Genest-Saint-Isle, France). They were kept in pairs with controlled humidity (55%) and temperature (22 °C), and with a light-dark cycle of 12 h. The rats were given food and water *ad libitum* and the animal facilities performed routine surveillance for pathogens upon arrival. The experiment was not controlled for the estrous cycle. The experiment adhered to the institutional guidelines for care and treatment of laboratory animals and was approved by the Regional Ethics Committee for animal experiments in Linköping, Sweden (ID1424). The study is reported according to the ARRIVE guidelines.

All rats underwent full transection of the right Achilles tendon, as described previously^13^. Briefly, during sedation with isoflurane gas (Forene, Abbot Scandinavia, Solna, Sweden) the plantaris tendon was removed, the Achilles tendon was transected in the middle of the tendon, and the skin was closed with two stitches (Supp Fig. S6). Subcutaneous injections of antibiotics (Engemycin, 25 mg/kg oxytetracycline) and analgesics (Temgesic, 0.045 mg/kg buprenorphine) were given pre-surgery. Rats received analgesics regularly after surgery for 48 h. At the end of each experiment, rats were anesthetized with isoflurane gas, and euthanized with carbon dioxide.

Altered loading was imposed through two mechanisms. To unload the right Achilles tendon, intramuscular Botox injections were given in the right calf muscles to induce plantar flexor muscle paralysis^24^. Briefly, during sedation with isoflurane gas, 3 U of Botox (Botulinum toxin, Allergan, Irvine, CA) was injected into the three calf muscles (gastrocnemius lateralis and medialis, and soleus muscle) at a dose of 1 U/muscle (0.02 ml/muscle). This procedure was done 4 days pre-surgery to reach full effect of Botox at the day of tendon injury. Additionally, the rats in the minimal loading group received a steel-orthosis around their right hindlimb directly after surgery, as previously described^24^. The metal boot was kept on all times until euthanasia, except during skin inspection, when rats were sedated with isoflurane and the boot was open.

### Histology

The right Achilles tendon together with a part of the calf muscle was harvested, incubated in sucrose solution (15% followed by 30%) and then optimal cutting temperature compound (OCT; VWR International AB, Spånga, Sweden, 00411243, OCT & 30% sucrose solution (1:1)). Tendons were placed in OCT, snap-frozen in liquid nitrogen, and stored at -80 °C. Frozen tendons were sectioned longitudinally (7 μm thickness) to reach the middle part of the tendon tissue. All tendons were first stained with hematoxylin & eosin (H&E) using standard protocols followed by immunofluorescent staining on consecutive slides. Sections were first hydrated in PBTD (PBS containing 0.1% Tween20 and 1% DMSO), fixed in 4% formaldehyde, and blocked with 5% normal goat serum for 2 h (Sigma Group, Malmö, Sverige; G9023). Primary polyclonal antibodies were incubated for 18 h at 4 °C, antibodies from rabbit were used against collagen 1, collagen 3, and elastin (collagen 1 ab34710 1:100, collagen 3 ab7778 1:50, elastin ab21610 1:100; Abcam, Cambridge, UK), and antibodies from mouse were used against CD31 (ab64543 1:200; Abcam, Cambridge, UK). Secondary antibodies were incubated for 2 h (goat anti-rabbit IgG A32740/donkey anti-mouse IgG A32744/goat anti-rabbit IgG A32731, Alexa Fluor Plus 594/594/488 Highly Cross-Adsorbed Secondary Antibody, Thermo Fisher Scientific, Rockford, USA; collagen 1 1:400, collagen 3 1:300, elastin 1:400, CD31 1:400). Lastly, tendon sections were counterstained with DAPI (4’,6-Diamidino-2-Phenylindole Dihydrochloride; 1:2000 diluted in PBTD; Thermo Fisher Scientific, Stockholm, Sweden; 62248) for 3 min followed by mounting (ProLong gold antifade reagent, Thermo Fisher Scientific, Stockholm Sweden; P36930). Kidney from rat was used as a positive control for collagen 1, and skin from rat was used as a positive control for collagen 3 and elastin.

Tissue sections were imaged under a microscope (DMi8, Leica Microsystems, Wetzlar, Germany, with a Hamamatsu Orca Flash 4 v3 sCMOS camera) where fluorescence was excited with a lumencor spectra X using 550 nm (secondary antibody Alexa Fluor 594), 470 nm (secondary antibody Alexa Fluor 488) or 385 nm (DAPI), and exposure time was held constant for each color channel regarding magnification and staining. Images were obtained from all tissue sections at both stump-callus interfaces and in mid callus, at the right, center and left side, of each staining (collagen 1, collagen 3 and elastin) with magnifications of 10x/0.30, 25x/0.95 (water) and 63x/1.20 (water) (Supp Fig. S7). Additionally, for one section per group (*n* = 1 per healing time, loading group and ECM matrix protein staining), the entire tendon callus was imaged in tile scan-mode with Leica DMi8 widefield (mosaic merge, 10% overlap). All images were adjusted to the negative control, where the primary antibody was omitted, to correct for unspecific antibody detection.

Due to various problems during sectioning and staining, 7 samples were excluded after the staining procedure, distributed in the following groups: 2 for collagen 1 (RL 1 week, FL 20 weeks), 3 for collagen 3 (ML 1 week, RL 1 week, RL 2 weeks), and 2 for elastin (RL 1 week, FL 3 weeks).

### Image analysis

All images were evaluated qualitatively in a blinded manner, and all mid, center images were analyzed quantitatively using in-house Matlab scripts to quantify collagen 1, collagen 3, and elastin reformation, cell properties (such as density, shape, and orientation), and blood vessel regeneration.

The image analysis steps were as follow:

1. For each original image, red (collagen 1, collagen 3, or elastin) and blue (DAPI) channels were split, and the data was converted from RGB to 8 bits images (Supp Fig. S8).
2. Collagen 1 and 3 (Supp Fig. S8A): To distinguish between forming fibers and blood vessels in images stained for collagen 1 or 3, a segmentation mask for blood vessels was created using a threshold value of 0.9 on the red channel image. The masked features were dilated of 8 pixels to ensure that the external rim of each blood vessel was included and then applied to the red channel image. Sequentially, collagen 1 or 3 staining areas were selected from the background using a threshold value of 0.12. *Calculations*: Mean intensity in the regions covered by collagen 1 or 3 was calculated from the masked images, generating an estimation of the amount of the specific extracellular matrix protein in each image. To estimate blood vessel density, total area covered by blood vessels were calculated from the masked area and normalized by total image area.
3. Elastin (Supp Fig. S8C): Blood vessels were not visible in images stained for elastin. However, bright dots due to unwashed dye were present. Therefore, to segment the regions stained for elastin, a mask for rounded objects with aspect ratio below 0.5 was created to exclude these dots in the analysis using a threshold value of 0.7. Next, a dilation of 5 pixels was performed, and the resulting mask was applied to the red channel image. Sequentially, areas stained for elastin were selected from the background using a threshold value of 0.2. *Calculations*: Mean intensity in the area covered by elastin was calculated form the masked images, generating an estimation of the amount of this extracellular matrix protein in each image.
4. Cell nuclei (Supp Fig. S8B): Cell nuclei were segmented from the blue channel that showed counterstaining of DAPI. This was done in tendons stained for collagen 1 by first masking the region of blood vessels, using the mask created for collagen 1, and then binarizing the images. Individual cells were identified and labelled. *Calculations*: Total area covered by cell nuclei were normalized to the total image area as a proxy to estimate cell density. To provide information about cell shapes the cell nuclei aspect ratio was obtained by fitting an ellipse to the cell nuclei and calculating the ratio between shortest and longest axes (1 = round, < 1 elongated cells). Cell organization was evaluated as the spread of cell nuclei orientations. The orientation of individual cells was calculated as the angle between the cell longest axis and the horizontal image plane. The histogram distribution of orientations was obtained, and the spread of the distribution was calculated as the full width at half maximum (FWHM).

Tendon diameter was measured in the central callus on the sectioned tendons on the glass slides. The diameter was used to normalize image analysis data to callus size as mechanical loading has a large impact on callus size.

### Statistical analysis

Statistical analysis was performed using GraphPad Prism 10.2.3. The effect of load levels and time-points were studied using a two-way ANOVA with the data from early healing (1-, 2- and 3-weeks post injury) followed by Tukey´s post hoc analysis for multiple comparisons for load differences at each time-point. The effect of time during late healing was analyzed in fully loaded animals (3-, 12- and 20-weeks post injury) with a one-way ANOVA followed by Tukey´s post hoc analysis for multiple comparisons.

## Electronic supplementary material

Below is the link to the electronic supplementary material.

Supplementary Material 1

## Data Availability

The data that support the findings of this study are in part available on Zenodo (https://doiorg/105281/zenodo10910680), and on request from the corresponding author.
