# Supplementary material for: Deprivation of loading during rat Achilles tendon healing affects extracellular matrix composition and structure, and reduces cell density and alignment
Source: Sci Rep. 2024 Oct 8;14:23380. doi: 10.1038/s41598-024-74783-w (PMC11461875; doi:10.1038/s41598-024-74783-w)
Supplement: Supplementary file 1 — Supplementary Material 1 [file 41598_2024_74783_MOESM1_ESM.pdf]

SUPPORTING INFORMATION

Deprivation of loading during early healing of rat Achilles tendons affects extracellular matrix composition and structure, and reduces cell density and alignment

Malin Hammerman, Maria Pierantoni, Hanna Isaksson, Pernilla Eliasson

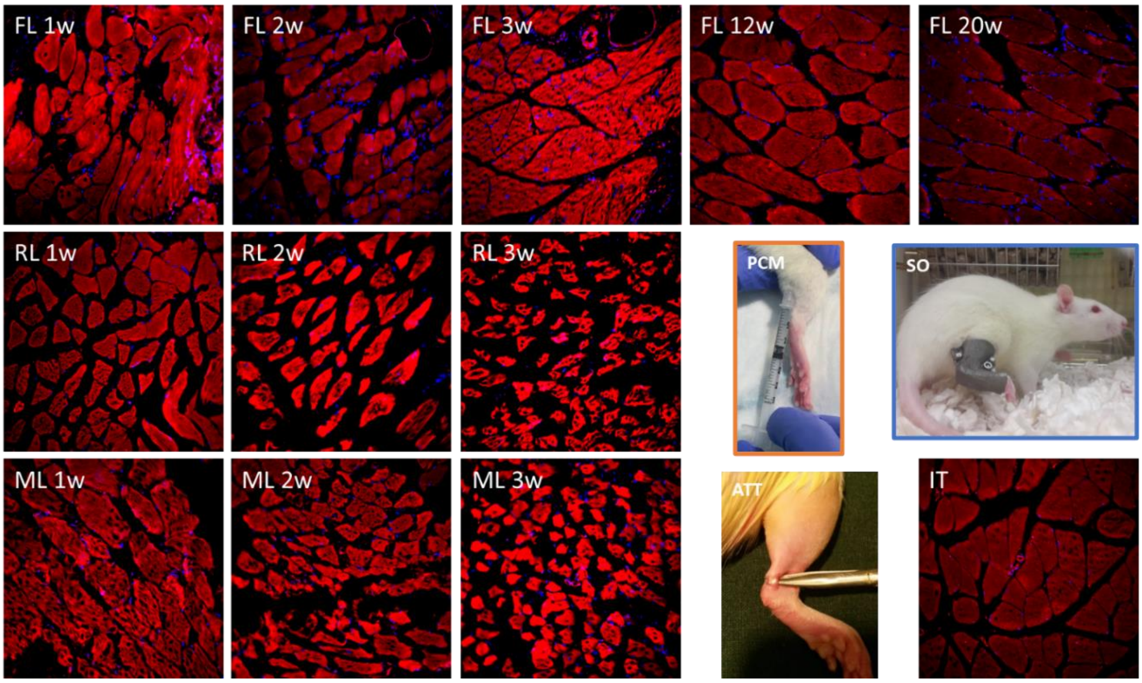

Figure S1. Cross-section of calf muscles. *Staning of elastin (red) and cell nuclei (blue) in calf muscles from rats in intact (IT) and healing tendons, which had been exposed to an Achilles tendon transection (ATT). Healing tendons were exposed to different loading conditions (ML=minimal loading - paralysis of calf muscle (PCM) and wearing a steel-orthosis (SO), RL=reduced loading (PCM), or FL=full loading) for 1, 2 or 3 weeks post-injury.*

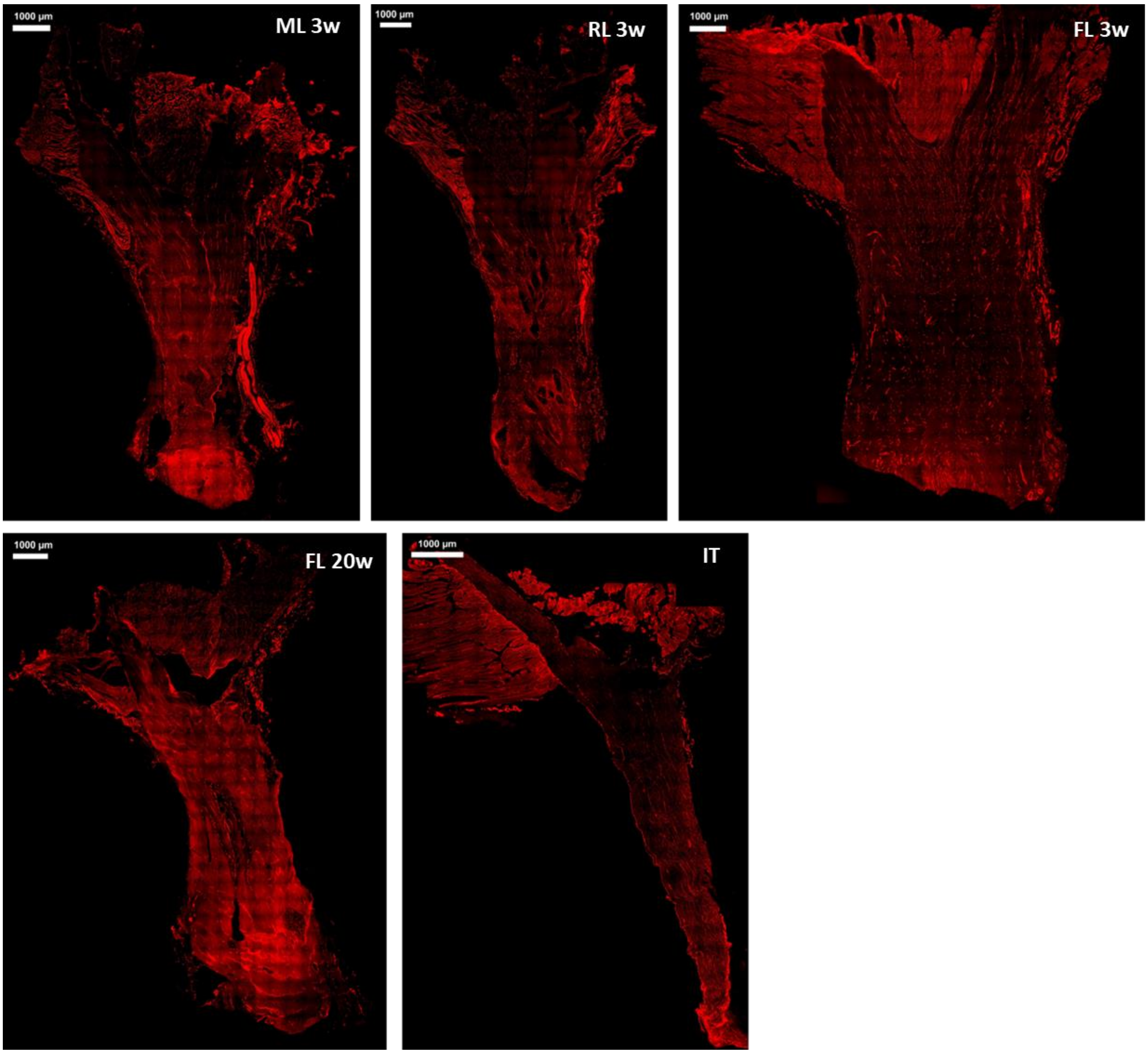

Figure S2. Overview tile images of complete tendons (Fig. S1: left) stained for collagen 1. *High resolution images were acquired at 3 weeks of healing for different loading conditions (ML: minimal loading, RL: reduced loading, or FL: full loading) and at 20 weeks for full loading and an intact tendon (IT) as reference. Scale bar 1000 µm. Full resolution images, with a possibility to zoom in and out, are available on Zenodo (https://doi.org/10.5281/zenodo.10910680 (1)).*

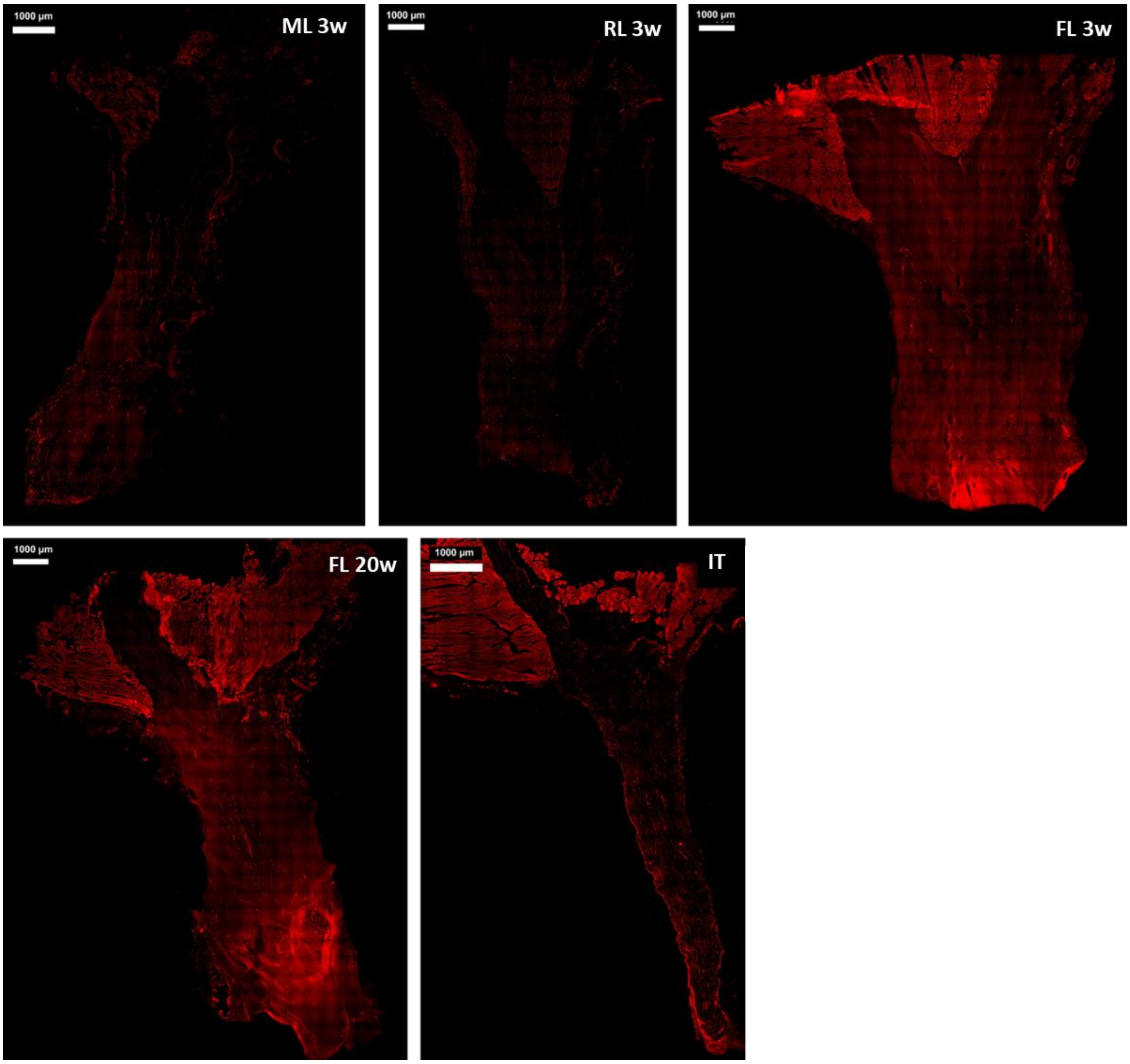

Figure S3. Overview tile images of complete tendons (Fig. S1: left) stained for collagen 3. *High resolution images were acquired at 3 weeks of healing for different loading conditions (ML: minimal loading, RL: reduced loading, or FL: full loading) and at 20 weeks for full loading and an intact tendon (IT) as reference. Scale bar 1000 µm. Full resolution images, with a possibility to zoom in and out, are available on Zenodo (https://doi.org/10.5281/zenodo.10910680 (1)).*

.

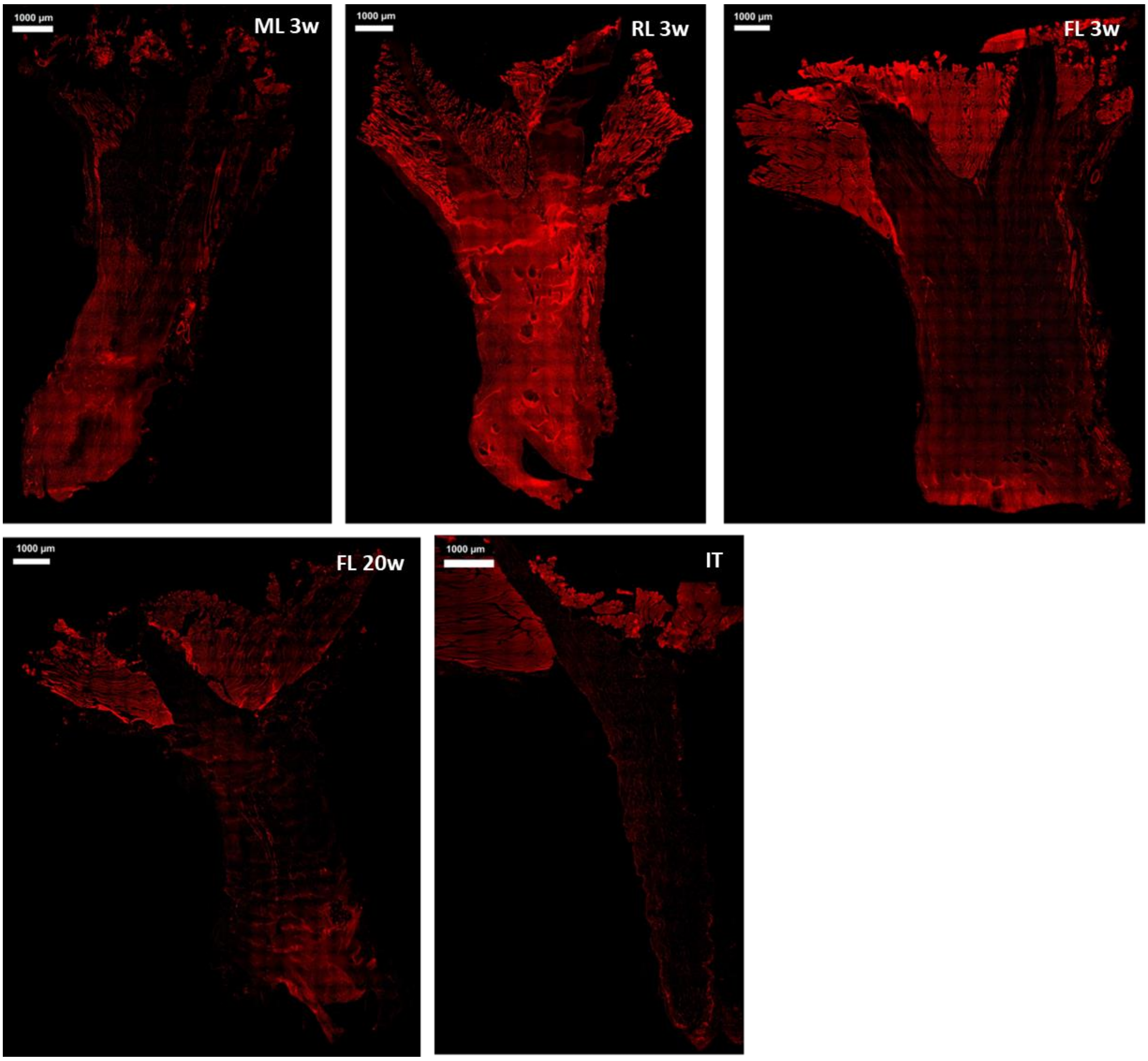

Figure S4. Overview tile images of complete tendons (Fig. S1: left) stained for elastin. *High resolution images were acquired at 3 weeks of healing for different loading conditions (ML: minimal loading, RL: reduced loading, or FL: full loading) and at 20 weeks for full loading and an intact tendon (IT) as reference. Scale bar 1000 µm. Full* resolution images, with a possibility to zoom in and out, are available on *Zenodo* (*https://doi.org/10.5281/zenodo.10910680 (1)*).

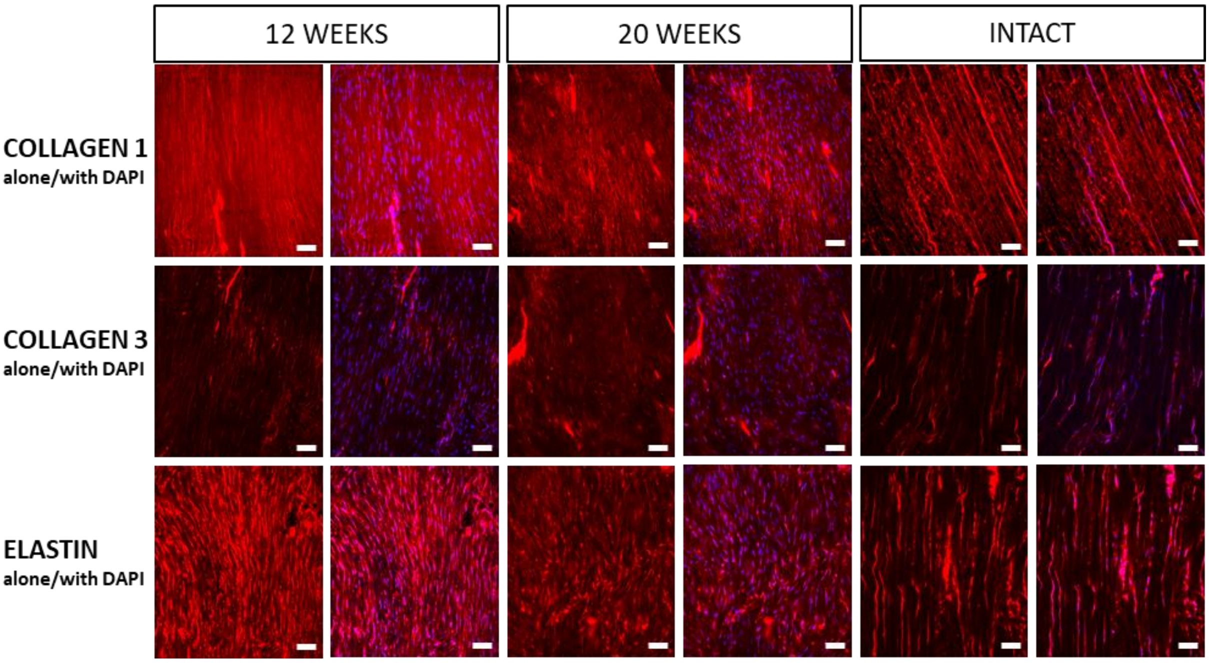

Figure S5. Intact and healing tendons at 12 and 20 weeks with brighter adjustments to clearly vizualize ECM distibution in these tendons. *Tendons are stained with antibodies against collagen 1, collagen 3, or elastin (red) and DAPI (blue). Images are taken in the middle of the tendon tissue. All tendons have been exposed to full loading. Scale bar 50 µm.*

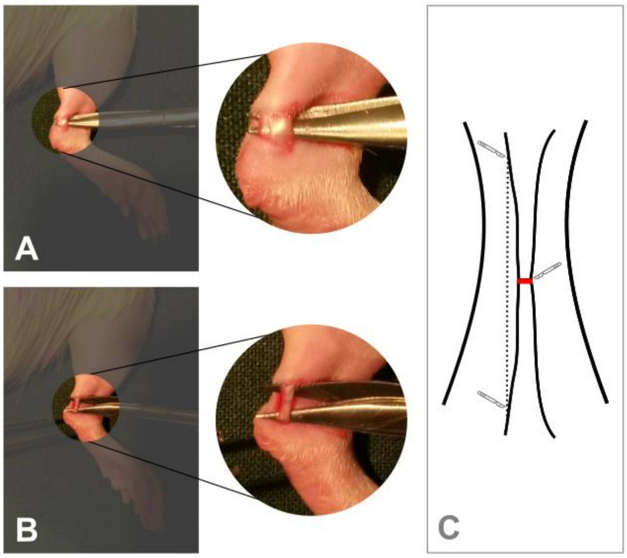

Figure S6: Figure of the surgical model for Achilles tendon transection. A) the Achilles and plantaris tendon complex were exposed through a small incision on the lateral side of the right hind leg. B) displays the portion of the plantaris tendon that was removed before the Achilles tendon was sectioned in the middle. C) provides a schematic illustration of the standard surgical procedure. The red line indicates the transverse cut made on the Achilles tendon, while the dotted line on the left shows the plantaris tendon that was removed. This image was adapted from Dietrich, 2017 (2).

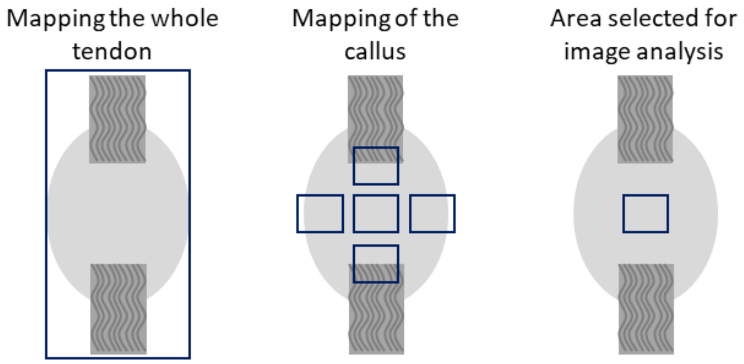

Figure S7. Schematic showing where images were acquired. *Left: The whole tendon was mapped in one tendon, for each time point, loading groups and proteins. Middle: Five higher magnification images were acquired in the callus for all time points, loading groups and proteins. It showed no systematic differences between the lateral side, the medial side, and in the center of the mid callus images. Right: Thus, the central image was selected for all quantitative image analysis.*

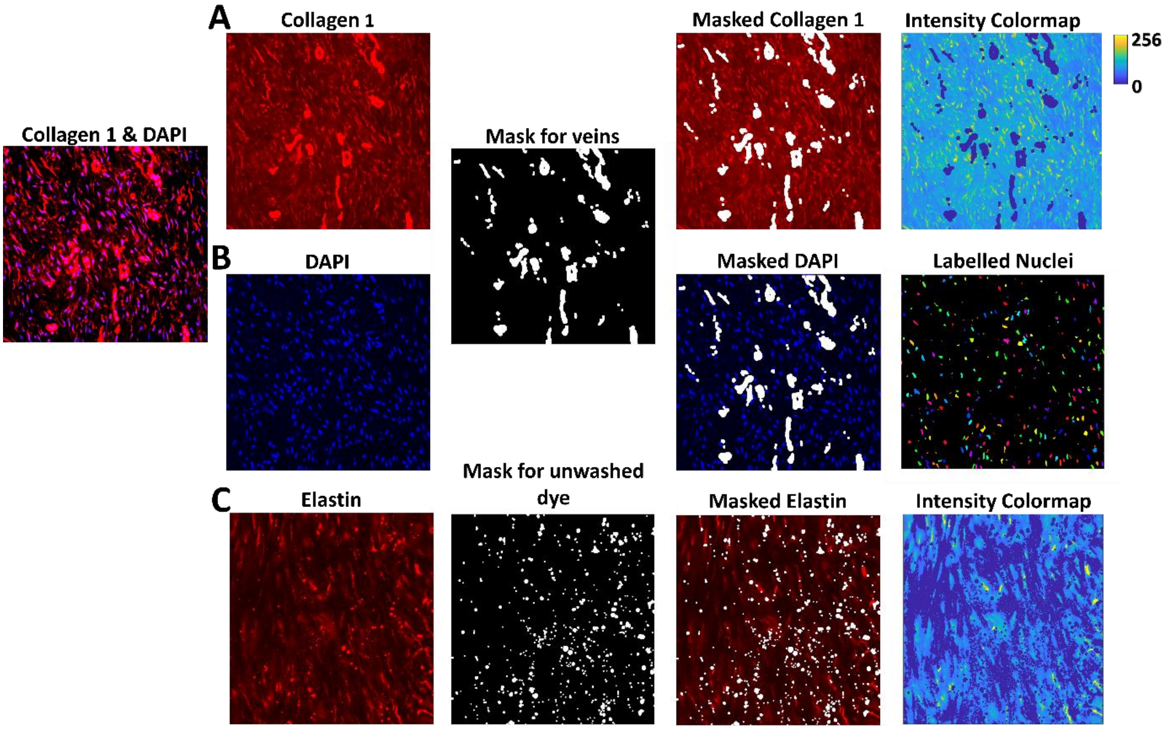

Figure S8. Image analysis pipeline for quantitative assessment of collagen 1 (A), cell nuclei (B), and elastin (C). *The original images were split in channels to consider extracellular matrix proteins and cells separately. After unwanted signal was masked out, collagen 1, collagen 3 and elastin were selected form the background, and intensities were calculated (colormap). Cells were labeled, and for each individual cell, properties such as shape and orientation were extracted.*

References

1. Hammerman M, Pierantoni M, Isaksson H, Eliasson P. Data description: Deprivation of loading during early healing of rat Achilles tendons affects extracellular matrix composition and structure, and reduces cell density and cell alignment. Zenodo https://doiorg/105281/zenodo10910680. 2024.
2. Dietrich F. Efeito do plasma rico em plaquetas no reparo do tendão de Aquiles em ratos: PONTIFÍCIA UNIVERSIDADE CATÓLICA DO RIO GRANDE DO SUL; 2017.
